# Multitrophic diversity effects of network degradation

**DOI:** 10.1002/ece3.2253

**Published:** 2016-06-21

**Authors:** Elizabeth Nichols, Carlos A. Peres, Joseph E. Hawes, Shahid Naeem

**Affiliations:** ^1^ Department of Biology Swarthmore College Swarthmore Pennsylvania 19081; ^2^ Department of Ecology University of São Paulo Sao Paulo 05508‐090 Brazil; ^3^ School of Environmental Sciences University of East Anglia Norwich Research Park Norwich NR47TJ U.K; ^4^ Animal and Environment Research Group Department of Life Sciences Anglia Ruskin University East Road Cambridge CB1 1PT U.K; ^5^ Department of Ecology, Evolution and Environmental Biology Columbia University New York New York 10027

**Keywords:** Biodiversity–ecosystem function, coextinction, dung beetles, fecal detritus, food webs, interaction networks, mammals, node loss, trophic interaction, tropical forest

## Abstract

Predicting the functional consequences of biodiversity loss in realistic, multitrophic communities remains a challenge. No existing biodiversity–ecosystem function study to date has simultaneously incorporated information on species traits, network topology, and extinction across multiple trophic levels, while all three factors are independently understood as critical drivers of post‐extinction network structure and function. We fill this gap by comparing the functional consequences of simulated species loss both within (monotrophic) and across (bitrophic) trophic levels, in an ecological interaction network estimated from spatially explicit field data on tropical fecal detritus producer and consumers (mammals and dung beetles). We simulated trait‐ordered beetle and mammal extinction separately (monotrophic extinction) and the coextinction of beetles following mammal loss (bitrophic extinction), according to network structure. We also compared the diversity effects of bitrophic extinction models using a standard monotrophic function (the daily production or consumption of fecal detritus) and a unique bitrophic functional metric (the proportion of daily detritus production that is consumed). We found similar mono‐ and bitrophic diversity effects, regardless of which species traits were used to drive extinctions, yet divergent predictions when different measures of function were used. The inclusion of information on network structure had little apparent effect on the qualitative relationship between diversity and function. These results contribute to our growing understanding of the functional consequences of biodiversity from real systems and underscore the importance of species traits and realistic functional metrics to assessments of the ecosystem impacts of network degradation through species loss.

## Introduction

The ongoing biodiversity crisis has spurred over 20 years of investigation into the impacts of species loss on ecosystem functioning and societal well‐being (Hooper et al. 2012; Naeem et al. 2012). A broad scientific consensus now holds that a greater intratrophic‐level diversity of genes, species, and functional traits is more efficient at capturing resources, contributes to greater biomass production (Cardinale et al. 2011), nutrient cycling, and decomposition rates (Hooper et al. 2012), and leads to the widely observed positive, saturating relationship between biodiversity and ecosystem functioning. The clearest evidence for these relationships comes from controlled manipulative experiments. However, the detection of clear and consistent associations between biodiversity loss and ecological function in complex natural systems remains elusive (Thébault and Loreau 2003), in part because diversity effects in real systems can be obscured by several factors.

First, existing research has largely focused on biodiversity losses within a single trophic level (i.e., monotrophic extinction; Duffy et al. 2007; Hillebrand and Matthiessen 2009), despite the fact that biodiversity loss invariably occurs across multiple trophic levels as a consequence of direct coextinction of dependent species (Koh et al. 2004) or indirect extinction through extinction cascades (Pimm 1980). The functional consequences of extinction across two or more trophic levels can be qualitatively different from those predicted by monotrophic studies (Thébault and Loreau 2003), as the species in different trophic levels affect function in both independent and interactive ways (Bruno et al. 2008), and because food web properties strongly influence the order and severity of secondary extinctions (Allesina et al. 2006; Borrvall and Ebenman 2006).

Second, the vast majority of diversity‐effect studies express ecological function as a monotrophic‐level process, such as the number of flowers visited by bee pollinators. However, many functions are inherently bitrophic in nature, as they represent the flux of energy or material between distinct food web components (Jax 2005). Examples include decomposition (where detritus produced by one trophic level is decomposed by another), pollination (where pollen produced by one trophic level is removed by another), predation, resistance to invasion, resistance to disease spread, nutrient cycling, and others (Gamfeldt et al. 2008). These functions are perhaps more realistically represented by metrics that incorporate the functional contribution of both trophic levels, such as the proportion of prey taken by predators, or the percentage of flowers produced by plants that are successfully pollinated by bees.

Third, while network topology is known to be a critical determinant of functional outcomes in multitrophic extinction (Thébault and Loreau 2003), it is seldom explicitly incorporated into biodiversity–ecosystem function models. Food web structure mediates the species interactions that can influence the order and probability of secondary species loss (Dyer and Letourneau 2003), as well as ecosystem function production (Montoya et al. 2003). The absence of explicit inclusion of network structure in studies of biodiversity loss across trophic levels implicitly assumes that all species are evenly connected across the interaction network (i.e., have identical linkage strength and density). An abundance of empirical data suggests this is unrealistic and that real ecological networks demonstrate a range of structures that strongly influence how primary extinctions may translate into secondary extinctions (Bascompte and Jordano 2007).

Despite the potential contribution of these three factors to increasing realism in BEF studies, we know of no study that has simultaneously integrated them with trait‐ordered scenarios of species extinction. For example, studies that examine the influence of nonrandom trait‐based extinction on ecological function have not incorporated explicit information on multitrophic extinctions, or network structure (Solan et al. 2006, Bunker et al. 2005; Bracken and Williams 2013). Multitrophic extinction studies that explicitly incorporate network structure have examined how extinction impacts network persistence, although not ecological function (Dunne et al. 2002b; Allesina and Bodini 2004; Allesina et al. 2006), or have done so without reference to species traits (Thébault and Loreau 2003). Other studies that assess the functional impacts of multitrophic extinction have done so without reference to network structure and often report conflicting directions and varying magnitudes of diversity effects. Even within a simple and similar aquatic system, Bruno et al. (2008) and Gamfeldt et al. (2005) found strong diversity effects of consumers (aquatic herbivores) on primary algal production, while Naeem et al. (2000) and Fox (2004a) found that consumer diversity had little effect on primary algal productivity. Gamfeldt et al. (2005) reported that consumer diversity increased consumer production, while Douglass et al. (2008) found no relationship between consumer diversity and consumer abundance or population growth rates. Finally, we know of no BEF study that has measured function as a bitrophic process that incorporates the functional contributions of both interacting trophic levels.

We suggest that some insight into the impacts of biodiversity loss from ecological systems may be gained by integrating these three components (multitrophic species extinction, bitrophic functions, and network structure) into trait‐ordered assessments of the functional consequences of extinction. We test this framework by examining the functional consequences of multitrophic species loss in a bitrophic fecal detritus system. While detrital pathways are a dominant component of most ecosystems (Moore et al. 2004), they have received little attention from existing biodiversity–ecosystem function research (Balvanera et al. 2006). This oversight matters, as the lack of compensatory responses of detritus to consumption suggests that the functional impacts of multitrophic species loss across “brown” and “green” world networks may be distinct (Srivastava et al. 2009).

We combined data on the abundance of fecal detritus producers (medium‐to‐large‐bodied tropical mammals) and fecal detritus consumers (Scarabaeine dung beetles) with trait‐based ecosystem function rates (daily feces production and consumption), and a producer–consumer network topology estimated from spatial co‐occurrence data. Dung beetles are a cosmopolitan group of fecal detritivores, whose incorporation of vertebrate feces into the soil layer during feeding and reproduction enhances nutrient cycling rates, contributes to early plant recruitment through secondary seed dispersal, and regulates the transmission of many gastrointestinal parasites of mammals (Nichols et al. 2008; Nichols and Gómez 2014). Their dependency on fecal detritus also places them at risk of cascading extinction following mammal decline (Nichols et al. 2013a). We simulated extinctions within individual (mono) trophic levels and contrasted their diversity effects with those produced by contingent species loss, across coupled (bi) trophic levels, where the sequence of secondary species loss was informed with data on network structure. We further explored how the use of either mono‐ or bitrophic measures of function, as well as the use of different trait‐based extinction scenarios, influenced results. To our knowledge, this is the first study that explores the functional consequences of multitrophic species loss through the explicit incorporation of information on network structure and species traits.

## Materials and Methods

### Field data

Data on mammal and dung beetle community structure were collected in 2009 along the Juruá River of western Brazilian Amazonia (State of Amazonas, Brazil) across a 100‐ha (1 km × 1 km) grid within the Uacari Sustainable Development Reserve (plot corners NW: −67.340339, −5.526063; SE: −67.32771, −5.527867). This grid consisted of twelve 1‐km transects, spaced 200 m apart. Dung beetle (Coleoptera: Scarabaeidae: Scarabaeinae) data were taken from pitfall traps placed at the intersection points between these transects, and at points along transects equidistant from each intersection point, for a total of 96 collection points. Pitfall traps followed a standardized design, with a receptacle (20 cm diameter, 15 cm depth) buried flush with the ground and baited with 20 g of fresh human dung, a standard broad‐spectrum attractant for Neotropical dung beetles. While some dung beetle species are highly specialized (Larsen et al. 2006), the majority have wide diet breadths and demonstrate consistent attraction to human dung‐baited traps (Marsh et al. 2013).

Captured beetle specimens were separated to species by a dung beetle taxonomist, following Vaz‐de‐Mello et al. (2011). Traps were collected after 48 h, during a single collection period in October 2009. Dung beetle body mass estimates were obtained by weighing between one and 30 captured individuals per species on a balance accurate to 0.0001 g after drying in a constant‐temperature oven at 60°C for 1 week. Data on medium‐to‐large‐bodied terrestrial and arboreal mammals were obtained through line‐transect surveys along the same transects as dung beetle data. Each 1‐km transect was surveyed between 0630 and 1030 h over a period of 4–5 consecutive rainless days by a previously trained field assistant from the nearest village, accompanied by an experienced biologist (see Hawes and Peres 2014 for more details), for a total of 3 months (August–October). Mammal species identity, group size, and location were recorded for each visual and/or acoustic encounter and paired with literature values of mean adult body mass (Emmons and Feer 1997). Elevation was recorded at each of the 96 sampling sites. Mammal, beetle, and elevation data were also simultaneously collected with identical methodology from a second, independent 100‐ha grid, located at a distance of 57 km from the first grid, within the adjacent Médio Juruá Extractive Reserve (plot corners NW: −67.13801, −5.03998; SE: −67.12651, −5.04571; Figure S1.

### Network construction

Inferring biotic interactions from geographic proxies such as co‐occurrence patterns is an increasingly common solution to the persistent lack of empirical species interaction observations, particularly for interactions that are cryptic or challenging to survey (Faust and Raes 2012; Sáyago et al. 2013; Morales‐Castilla et al. 2015). This inference approach is most likely to generate plausible hypotheses about interactions when (1) forbidden interactions can be detected and excluded; and (2) information on co‐occurrence or distribution can be complemented by an analysis of the species–environment relationships for both sets of potentially interacting species (Morales‐Castilla et al. 2015).

We used the spatially explicit field data described above to estimate a beetle–mammal interaction network based on an environmentally constrained null model approach to estimate significant spatial co‐occurrence between mammal–beetle species pairs. In unconstrained null models, either the number of species occurrences or both species occurrences and site richness are maintained constant while incidence values are randomized across the matrix (Gotelli 2000). This implicitly assumes equal probability of occurrence colonization across sites, ignoring the possibility that similar co‐occurrence patterns could be generated by shared habitat associations, historical processes, or dispersal abilities (Peres‐Neto et al. 2001). Instead, an environmentally constrained approach informs the null model building process with site‐specific probabilities of occurrence for each species–site combination, using estimated species–environmental relationships (Peres‐Neto et al. 2001).

We used a key environmental parameter (elevation) and logistic regression to estimate site‐specific occurrence probabilities for all mammals and a subset of common dung beetles (density >0.1 individual ha^−1^) across all 96 sampling sites. If an individual mammal was observed within 50 m of one of the 96 sampling sites used to census dung beetles, it was scored as present for that sampling site. We first tested each individual species model for residual spatial autocorrelation with Moran's I test, using a weights matrix calculated from nearest neighbor distances (set to 100 m for dung beetles, and 300 m for mammals). We then transformed each individual site probability into relative probabilities by dividing each site‐specific probability by its respective column sum. To do this, we adopted the Ct‐RA2 approach of Peres‐Neto et al. (2001) to assign a value of 1.0 to all sites where a presence was observed and used this probability matrix to generate a new random‐constrained matrix of presence/absence values for dung beetle and mammal species across all 96 sites. Using this constrained presence/absence matrix, we estimated the positive co‐occurrence between all mammal and beetle species pairs by contrasting the *T*‐score statistic (*T*) from the observed and null models (using 1999 iterations), while maintaining a constant total number of species occurrences between observed and simulated communities. We defined species interactions as those for which *T*‐scores were positively significantly different from zero, based on a one‐sided test of the *z*‐value statistic. As most dung beetle species are feces generalists, we further assumed that forbidden dung beetle–mammal feces interactions were unlikely. We characterized the complexity of network structure as link density (the average number of links per species in a network; *L*/*S*), and connectance (the ratio of realized to potential interactions among *S* species in a network; *L*/*S* ^2^). For each individual trophic level within a network, we also calculated species‐level connectance (the ratio of realized to possible interactions with the opposing trophic level).

### Extinction simulations

We used the species present in the resulting bipartite interaction network as our intact community of fecal detritus producers and consumers. We modeled species loss from this community in two ways – by simulating the independent loss of producer or consumer species diversity (monotrophic extinction models), and by simulating primary producer loss and contingent secondary consumer loss (bitrophic extinction models), by first simulating producer species loss, then removing any subsequently unconnected consumer species in accordance with network structure.

For both mono‐and bitrophic models, species loss was simulated as (1) random; (2) proportional to body mass; or (3) inversely proportional to abundance. Larger body mass is thought to proxy for higher extinction risk for both dung beetles and mammals, via association with lower intrinsic rates of natural increase, longer generation time, lower population densities, and size‐biased hunter selectivity for mammals (Robinson and Redford 1986; Jerozolimski and Peres 2003; Nichols et al. 2013b). Extinction risk is typically high for rare species (i.e., with low local abundance), as small populations are more vulnerable to environmental and demographic stochasticity and tend to have narrow geographic distributions and specialized foraging guilds, compounding species risk (Olden et al. 2008).

### Function estimation

To compare the expected functional consequences of extinction using different functional metrics, we expressed function as both a monotrophic and bitrophic process. We estimated monotrophic function as the daily total amount of fecal detritus that could be produced by the mammal community (*F*_p_) or removed by the beetle community (*F*_r_), and bitrophic function as the proportion of fecal biomass produced by mammals that is consumed by beetles (*F*_r_ */F*_p_).

To estimate *F*_p_, we combined literature values of mammal body mass (*m*_*i*_) with field data on abundance (*N*_*i*_) across the 100‐ha study grid, and an allometric relationship between mammal body mass and fecal production (Blueweiss et al. 1978), and summarized these values across the mammal species remaining in a given simulation of species loss as:

(1) $$F_{p}=\sum_{i}^{j}\left(0.85\times {m_{i}}^{-0.37}\right)\times N_{i}m_{i}$$

To estimate *F*_r_, we combined field data on beetle biomass (*b*_*i*_) across the 100‐ha study grid (*N*_*i*_), and an estimated relationship between beetle biomass and feces removal rate modified from Horgan (2005), and summarized these values across all beetle species remaining in a given simulation of species loss as:

(2) $$F_{r}=\sum_{i}^{j}12.49+33.48\times b_{i}$$

To assess the relative influence of extinction on function and to simplify contrasting diversity effects across different function metrics, we normalized all measures of function by dividing by their respective maximum values within a given extinction scenario.

For monotrophic extinction models, we calculated *F*_p_ *and F*_r_ at each level of species loss (for mammals and beetles, respectively) in each run. For the bitrophic extinction models, we explored diversity effects in two ways. First, we calculated *F*_r_ at each level of secondary (i.e., dung beetle) species loss, to enable a direct investigation of the functional consequences of the inclusion of network structure. We did not calculate *F*_p_ in a similar bitrophic extinction model for producer extinction, as this is trivially identical to the monotrophic extinction scenario described above. Second, we calculated the bitrophic function (*F*_r_/*F*_p_) at each level of both primary (producer) and secondary (consumer) species loss, to explore the sensitivity of extinction to this more realistic measure of ecological function. For all models, we assumed a lack of compensatory dynamics, given the weak evidence for biomass or abundance compensation in perturbed Neotropical mammal and dung beetle communities (Peres and Dolman 2000; Nichols et al. 2013a).

### Statistical analysis

We assessed the basic relationships between species' traits (body mass and rarity) and observed occupancy across the 100‐ha plot (i.e., number of sampling sites occupied) with linear regression. For each extinction (mono‐ and bitrophic) and trait scenario (null, rarity, and body mass), we modeled the relationship between ln species richness and mean function (*F*_p_, *F*_r_, or *F*_r_/*F*_p_) with a beta regression – a generalization of a logit model ideal for situations where the response is continuous on the interval (0,1) (Cribari‐Neto and Zeileis 2010). We compared the relative strength of diversity effects between trait‐based extinction scenarios by calculating the ratio of trait scenario slopes to random (null) scenario slopes. Finally, for each scenario, we estimated the function at “catastrophic” species loss – where only a single producer or consumer species remained.

We explored the relationships between network complexity (linkage density and connectance) and function (*F*_r_/*F*_p_), with beta regression. Finally, given that cascading species extinctions in food webs are thought to be relatively resistant to the impacts of random species loss (error resistant) but sensitive to the removal of well‐connected nodes (attack prone; Albert et al. 2000; Dunne et al. 2002a), we were interested in positive associations between species‐level connectance and species' traits that correlate positively with extinction risk. To explore this, we used beta regression to model the relationship between species‐level connectance and species' trait values, as well as occupancy. All analyses were conducted in the R Statistical Environment (R Development Core Team 2011).

## Results

**Figure 1:**
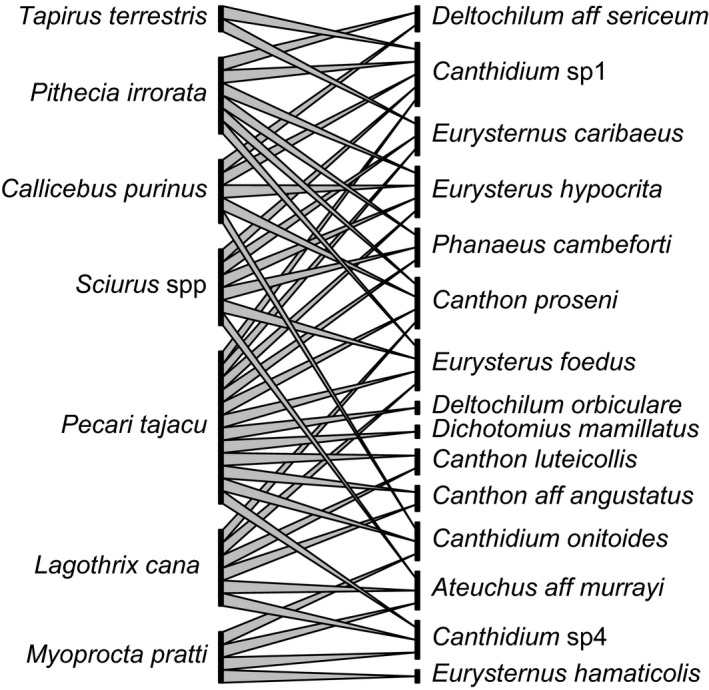
Dung beetle–mammal interaction network, estimated from spatially explicit co‐occurrence data from the western Brazilian Amazon. Overall network size (*S*) = 22 (15 consumer and seven producer species), average number of links per species (*L/S*) = 1.86, and proportion of possible links among *S* species that are actually realized (*L/S* ^2^) = 0.39.

Of the 102 dung beetle species and nine mammal species originally sampled across the 100‐ha grid, we estimated significant co‐occurrence between a total of 15 beetle and seven mammal species, which were included in subsequent analyses (Fig. 1). Body mass and total abundance across the 100‐ha plot ranged widely for both groups (beetles: mean 0.127 ± 0.12 g, range 0.008–0.46 g; 71.46 ± 79.15 individuals, range 11–323; mammals: mean 8.29 ± 6.70 kg, range 2–21 kg; 5.85 ± 4.67 individuals, range 1–12).

Extinction risk varied widely across fecal detritus consumers and producers, both when extinction probability was assumed proportional to rarity (consumers: 0.031 ± 0.028, range 0.003–0.091; producers: 0.357 ± 0.332, range 0.083–0.99) or proportional to body mass (consumers: 0.161 ± 0.158, range 0.011–0.585; producers: 0.57 ± 0.306, range 0.143–0.99). Values of body mass and abundance as well as occupancy were uncorrelated for mammals (all *P* > 0.5), while dung beetle abundance positively correlated with occupancy (*b* = 3.68, *t*_13_ = 4.77, *P = *0.0004).

The final ecological interaction network included an average of 5.71 connections per mammal species, and 2.73 per beetle species (Fig. 1). Across the entire network, the realized proportion of possible links (*L*/*S* ^2^) was 0.39, while linkage density (*L*/*S*) was 1.86. Under simulated scenarios of catastrophic mammal species loss (i.e., when a single mammal species persisted), more beetle species remained connected when mammal extinctions were ordered by body mass (mean 6.13 beetle species ± 2.50) than by rarity (mean 5.70 beetle species ± 2.16). The number of dung beetle species associated with each mammal species was unrelated to mammal occupancy (*P* > 0.5), while the number of mammal species links estimated for each beetle species was positively associated with beetle occupancy across the 100‐ha grid (*b* = 0.023, *z*_3_ = 2.46, *P = *0.014). We obtained qualitatively similar results for network size, average beetle species richness following catastrophic mammal loss, *L/S* ^2^, and *L/S* using independent data from a second 100‐ha grid collected at the same time, and subject to the same interaction network estimation (SOM, Fig. 1).

**Figure 2:**
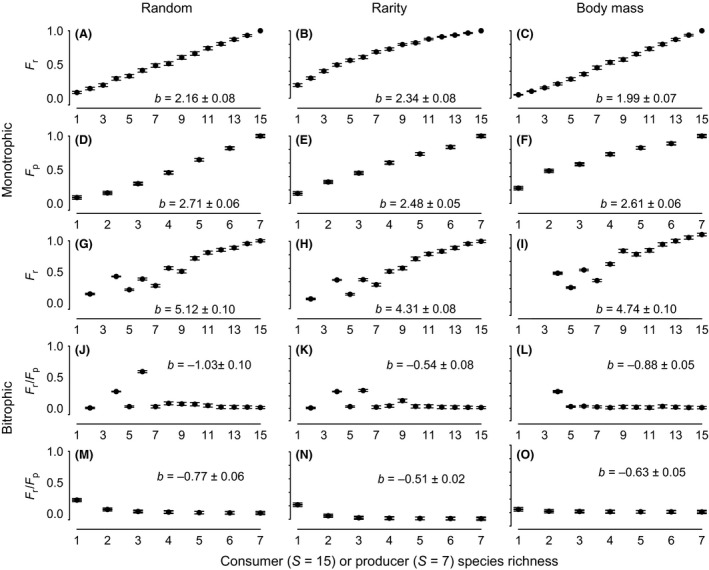
Simulated influence of biodiversity on fecal detritus production and removal rates in the western Brazilian Amazon. The intact communities contained seven detritus producer mammal species and 15 detritus consumer dung beetle species. Extinction was simulated both within individual trophic levels (monotrophic) and across trophic levels (bitrophic), where producer extinction was propagated to consumers according to network structure (see Fig. 1). For both monotrophic (A–F) and bitrophic (G–O) species loss, extinction was simulated as random (random), inversely proportional to observed species abundance (rarity) or proportional to body mass (body mass). Function in the monotrophic extinction models was calculated as the normalized daily rate of detritus production by the mammal community (*F*_p_) or detritus consumption by the beetle community (*F*_r_). Function in the bitrophic extinction models was calculated both as monotrophic (*F*_r_ as above) and bitrophic (the proportion of detritus produced by mammals that is consumed by beetles, *F*_r_/*F*_p_). All panels show mean and 95% confidence interval of mean function (*F*_r_, *F*_p_ *, or F*_r_/*F*_p_). Insets denote slope and 95% confidence interval.

We found that both producer and consumer extinctions in monotrophic models were associated with a decline in monotrophic function (*F*_p_ and *F*_r_), regardless of the trait‐ordered extinction scenario (Fig. 2A–F). For both producers and consumers, body mass‐ordered extinctions drove the strongest relationship between ‐ diversity and *F*_r_, as well as the highest estimated rates of *F*_r_ after catastrophic species loss (Table 1).

**Table 1:** Functional consequences of monotrophic or bitrophic extinction from a fecal detritus producer–consumer network

Trophic model	Species richness	Function type	Trait scenario	*F* at *S* = 1	Relative BEF slope	Pseudo *R* ^2^
Mono	Consumer	*F*_r_	Rarity	0.562	0.579	0.798
			Body mass	1.081	1.090	0.871
Mono	Producer	*F*_p_	Rarity	0.645	0.666	0.888
			Body mass	0.985	0.987	0.745
Bi	Consumer	*F*_r_	Rarity	0.755	0.753	0.897
			Body mass	0.800	0.798	0.853
Bi	Producer	*F*_r_/*F*_p_	Rarity	1.127	0.900	0.320
			Body mass	1.353	0.589	0.427
Bi	Consumer	*F*_r_/*F*_p_	Rarity	2.608	0.658	0.132
			Body mass	2.421	0.747	0.251

*Trophic model* denotes whether extinction was modeled within a single trophic level (monotrophic) or as contingent species loss propagated from producers to consumers (bitrophic). *Species richness* denotes the trophic level (producer or consumer) against which the relationship with function was evaluated. *Function type* is the functional measure used: either monotrophic (daily feces removal rate *F*_r_ and daily feces production rate *F*_p_) or bitrophic (proportion of feces produced that are removed *F*_r_/*F*_p_). The relationship between species richness and function was evaluated from a fitted beta regression model (mean function~ln species richness) with a logit link function. “*F* at *S *=* *1” is the estimate of function at one remaining species of the mammal (producer) or dung beetle (consumer) community, relative to the null model of random species extinction. *Relative BEF slope* is the ratio of the slope from each trait‐based extinction model to a random extinction (null) model and illustrates the relative strength of the BEF relationship for each trait‐based scenario relative to random. *Pseudo R* ^2^ is the proportion of variability explained by each fitted BEF model.

Similarly, we found that consumer extinctions in bitrophic extinction models (i.e., where primary extinctions of producers drove contingent secondary consumer extinctions) also resulted in monotrophic function decline (i.e., *F*_r_) across all trait‐based extinction scenarios (Fig. 2G–I), with body mass‐driven extinction of consumer species leading to both slightly stronger overall diversity effects and higher predicted function after catastrophic consumer species extinction (Table 1).

In contrast, when we represented function as a bitrophic process (i.e., as *F*_r_ */F*_p_), bitrophic extinction models predicted a slight increase in function with declining diversity, across all extinction scenarios and for both consumers (Fig. 2J–L) and producers (Fig. 2M–O). For these models, the influence of species traits differed for producers and consumers. Rarity‐ordered extinctions produced stronger diversity effects associated in producer species, yet predicted a lower level of remaining function following catastrophic producer extinction (Table 1). Body mass‐ordered extinctions produced stronger diversity effects associated in consumer species, yet predicted a lower level of remaining function following catastrophic consumer extinction (Table 1).

At the level of the interaction network, network structure and ecological function were strongly correlated across all bitrophic extinction models, regardless of extinction scenario. Function (*F*_r_ */F*_p_) was associated positively with network connectance (random: *b *= 2.62, *z*_698_ = 11.28, *P* < 0.0001; body mass: *b *= 1.54, *z*_698_ = 16.15, *P* < 0.0001; rarity: *b *= 2.27, *z*_698_ = 10.15, *P* < 0.0001), and negatively with linkage density (random: *b *= −1.04, *z*_698_ = −8.22, *P* < 0.0001; body mass: *b *= −0.812, *z*_698_ = −12.84, *P* < 0.0001; rarity: *b* = −0.72, *z*_698_ = −6.06, *P* < 0.0001).

At the level of individual trophic levels, we found some evidence for a relationship between species' traits and species‐level connectance. Smaller and more abundant dung beetles were connected to a higher proportion of the available mammal species (body mass: *b *= −3.22, *z*_3_ = −2.27, *P* = 0.023; rarity: *b *= 0.004, *z*_3_ = 1.98, *P* = 0.047). Less rare mammals were also connected to a higher proportion of the available dung beetle species (rarity: *b *= 0.13, *z*_3_ = 2.47, *P* = 0.013). Mammal body mass was unrelated to connectance (all *P* > 0.1).

## Discussion

These results provide the first demonstration, to our knowledge, of diversity effects in a multitrophic system where species traits and network structure are taken into consideration. We demonstrated similar monotrophic‐level diversity‐effect patterns to those widely observed in standard biodiversity–effect studies, independent of extinction scenario (i.e., random or trait based; Fig. 2A–F). Further, these same relationships were replicated even under bitrophic extinction, when consumer extinction was contingent on producer extinction (Fig. 2G–I). However, when the same bitrophic extinction was modeled with a more realistic bitrophic function of (*F*_r_ */F*_p_), models showed a negative relationship between diversity and function (Fig. 2J–O).

This marked reversal in the direction of biodiversity–ecosystem function relationships was exposed only when we integrated both components of function into a whole system response, resulting in higher estimated function rates at lower levels of species richness. In our system, the minimum daily detritus production of a mammal community following catastrophic extinction (i.e., a single mammal species) was 65.9 g. In contrast, the dung beetle community associated with that single mammal species was estimated to be able to consume a minimum of 2497 g feces day^−1^. This disproportionate effect of extinction on fecal detritus production, rather than consumption, was qualitatively robust to different trait‐based drivers of extinction. The vagility and active foraging strategies of dung beetles imply that some species may adjust to low‐resource availability by simply relocating to higher resource areas, suggesting that such localized “mismatches” between fecal detritus production and consumption may be sustainable in real communities (Nichols et al. 2013b). Similar spatial heterogeneity in function supply and demand may be common for many multitrophic functions that are mediated by mobile organisms (Tylianakis et al. 2008) and suggests that the functional consequences of biodiversity loss need to be accounted for independently within interacting trophic levels.

The differential effects of trait‐based extinctions on ecosystem function we observed are attributable in part to the different ways traits associate with the degradation of network structure. For example, we found that smaller beetles tended to be connected with more mammal species, potentially suggesting a size bias in resilience to local mammal extinction. Additionally, when we modeled beetle extinction as contingent upon mammal loss, we observed a diversity‐effect threshold, where detritus consumption both abruptly declined and became more variable following the removal of approximately 7–9 beetle species (Fig. 2G–I). This threshold was most visible when beetles were removed in descending order of body mass (Fig. 2I). Such a size‐biased diversity‐effect threshold is consistent with the observation that larger dung beetles are both disproportionately significant consumers of fecal detritus, and sensitive to habitat degradation (Larsen et al. 2005). While body size generally correlates with abundance and other key species traits related to persistence (Woodward et al. 2005), we found no such relationships for the mammal species sampled across the study plot.

Network structure was clearly associated with ecosystem function – which varied positively with network connectance, and negatively with linkage density. This difference likely stems from the numerical result of divergent relationships between species richness and connectance (negative when the ratio of realized to potential links in a food web decreases with increasing web size) and linkage density (positive when the average number of links per species increases in larger webs).

One general limitation of network analyses is that estimates of network size and structure are sensitive to sampling completeness, as well as to shifting temporal patterns of plant phenology that have cascading influences on localized mammal (Hawes and Peres 2016), and therefore dung beetle community composition. Although our estimates of network size (*S*) and complexity (*C*) could be improved through the use of additional plots and/or longer‐term sampling, our observation that mono‐ and bitrophic functions may show opposite responses to species loss is unlikely to be affected by additional sampling. To explore the robustness of this approach, we calculated a second consumer–producer interaction network using independent co‐occurrence data taken from a second 100‐ha grid within the same study region, sampled identically and within the same season, and which yielded a qualitatively similar network in all respects Figure S1. While the simplicity of our bitrophic detrital web and models of extinction may limit extrapolation to more complex systems (Duffy et al. 2009), simplified networks frequently provide important basic insights while reducing the intractability of larger, more complex systems (Holt and Loreau 2002). Our use of the bitrophic fecal detritus‐based system has the additional advantage over more complex systems in its lack of density‐dependent interactions and complex feedbacks that define bottom‐up and top‐down interactions in the plant world (Srivastava et al. 2009). Finally, we note that some diversity‐effect models include both direct effects (e.g., consumption) and indirect effects (e.g., niche expansion or numerical compensation following species loss) as indirect effects, which may buffer the functional consequences of species loss. Our decision to not include such effects reflects the current understanding about biomass and abundance compensation for these two taxonomic groups in the Neotropics, although we caution against extrapolating these results to other biogeographic regions where density compensation by open‐habitat affiliate species into degraded areas seems to be common (e.g., Nichols et al. 2013a).

Broadly, our findings point to four important issues in biodiversity and ecosystem functioning research. First, we have shown that the functional consequences of biodiversity loss are qualitatively similar between monotrophic and multitrophic systems. Second, our study illustrates that the choice of functional metrics (in this study, *F*_r_, *F*_p_, or *F*_r_/*F*_p_) to describe function can strongly influence our interpretation of diversity effects. Third, our results reinforce the importance of considering different trait‐based extinction scenarios as models for biodiversity loss as well as random loss to determine the ecosystem consequences of biodiversity loss. Fourth, our findings reinforce that network approaches can be valuable tools for predicting the consequence of environmental change on ecosystem function (Galiana et al. 2014). Given the prevalence of multi trophic networks in ecological systems, our current understanding of the effects of biodiversity on ecosystem functioning, derived predominantly from monotrophic studies (see recent review by Tilman et al. 2014), remains relatively incomplete.

## Conflict of Interest

None declared.

## Supporting information


**Figure S1.** A second dung beetle‐mammal interaction network, estimated from spatially explicit co‐occurrence data from the western Brazilian Amazon, from an independent, and simultaneously collected dataset (see Methods). Overall network size (*S*) = 22 (17 consumer and five producer species), average number of links per species (*L/S*) = 1.27, and proportion of possible links among S species that are actually realized (*L/S* ^2^) = 0.32. Despite their relative proximity, several key taxonomic differences, associated with parapatric species replacements across the Rio Jurua riverine barrier, exist between the mammal fauna found in this plot and the plot sampled in Figure 1 (see text). Notably, the monk saki monkey (*Pithecia monachus*; this figure) and bald‐faced saki monkey (*P. irrorata*; Fig. 1) occur on the left and right banks of the Rio Jurua, respectively.
