# Supplementary figures and images for: Multitrophic diversity effects of network degradation
Source: Ecol Evol. 2016 Jun 21;6(14):4936–46. doi: 10.1002/ece3.2253 (PMC4979718; doi:10.1002/ece3.2253)

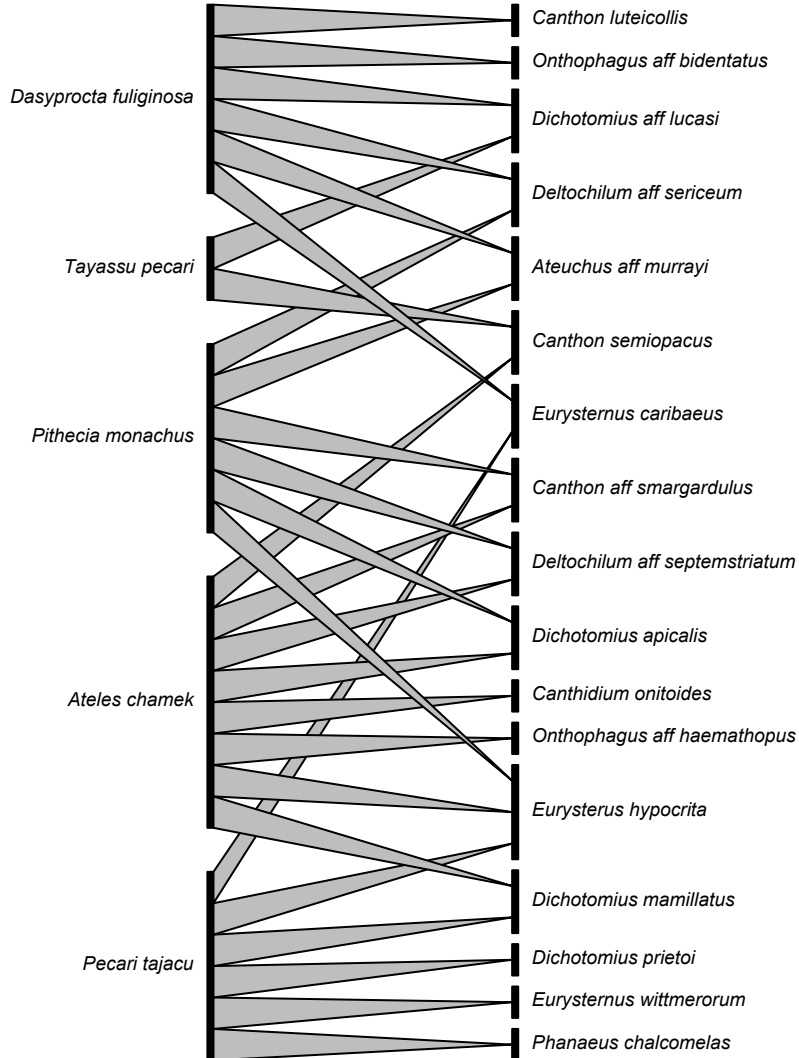

Supplement: Supplementary file 1 — Figure S1. A second dung beetle‐mammal interaction network, estimated from spatially explicit co‐occurrence data from the western Brazilian Amazon, from an independent, and simultaneously collected dataset (see Methods). Overall network size (S) = 22 (17 consumer and five producer species), average number of links per species (L/S) = 1.27, and proportion of possible links among S species that are actually realized (L/S 2) = 0.32. Despite their relative proximity, several key taxonomic differences, associated with parapatric species replacements across the Rio Jurua riverine barrier, exist between the mammal fauna found in this plot and the plot sampled in Figure 1 (see text). Notably, the monk saki monkey (Pithecia monachus; this figure) and bald‐faced saki monkey (P. irrorata; Fig. 1) occur on the left and right banks of the Rio Jurua, respectively. [file ECE3-6-4936-s001.pdf]
